# The Role of Genetic Ancestry as a Risk Factor for Primary Open-angle Glaucoma in African Americans

**DOI:** 10.1167/iovs.62.2.28

**Published:** 2021-02-19

**Authors:** Brian S. Cole, Harini V. Gudiseva, Maxwell Pistilli, Rebecca Salowe, Caitlin P. McHugh, Michael C. Zody, Venkata R. M. Chavali, Gui Shuang Ying, Jason H. Moore, Joan M. O'Brien

**Affiliations:** 1 Institute for Biomedical Informatics, University of Pennsylvania, Philadelphia, Pennsylvania, United States; 2 Scheie Eye Institute, University of Pennsylvania, Philadelphia, Pennsylvania, United States; 3 New York Genome Center, New York City, New York, United States

**Keywords:** ancestry, glaucoma, primary open-angle glaucoma, African Americans, genetics

## Abstract

**Purpose:**

POAG is the leading cause of irreversible blindness in African Americans. In this study, we quantitatively assess the association of autosomal ancestry with POAG risk in a large cohort of self-identified African Americans.

**Methods:**

Subjects recruited to the Primary Open-Angle African American Glaucoma Genetics (POAAGG) study were classified as glaucoma cases or controls by fellowship-trained glaucoma specialists. POAAGG subjects were genotyped using the MEGA Ex array (discovery cohort, *n* = 3830; replication cohort, *n* = 2135). Population structure was interrogated using principal component analysis in the context of the 1000 Genomes Project superpopulations.

**Results:**

The majority of POAAGG samples lie on an axis between African and European superpopulations, with great variation in admixture. Cases had a significantly lower mean value of the ancestral component q0 than controls for both cohorts (*P* = 6.14^–^^4^; *P* = 3^–^^6^), consistent with higher degree of African ancestry. Among POAG cases, higher African ancestry was also associated with thinner central corneal thickness (*P* = 2^–^^4^). Admixture mapping showed that local genetic ancestry was not a significant risk factor for POAG. A polygenic risk score, comprised of 23 glaucoma-associated single nucleotide polymorphisms from the NHGRI-EBI genome-wide association study catalog, was significant in both cohorts (*P* < 0.001), suggesting that both known POAG single nucleotide polymorphisms and an omnigenic ancestry effect influence POAG risk.

**Conclusions:**

In sum, the POAAGG study population is very admixed, with a higher degree of African ancestry associated with an increased POAG risk. Further analyses should consider social and environmental factors as possible confounding factors for disease predisposition.

POAG is an insidious neurodegenerative disease of the optic nerve that causes progressive vision loss.^1^ This disease is a leading cause of irreversible blindness worldwide, with global projected prevalence of up to 80 million cases by 2040.^2^ African Americans are four to five times more likely to be diagnosed with POAG than European Americans^3^^,^^4^ and present with the disease at a younger age with more severe and rapidly progressing symptoms.^5^^–^^7^ This population is 15 times more likely to become visually impaired from POAG than European Americans.^8^

Many twin and family history studies have confirmed that POAG has a strong genetic component,^9^^–^^12^ yet more than 90% of its genetics remain unexplained.^13^ The remaining 10% is composed of variants of small individual effect sizes, mostly discovered in populations of European or Asian descent.^13^^–^^18^ These variants often have a reduced or unknown role in individuals of African descent, suggesting that these individuals may have different susceptibility alleles for POAG than other ethnic groups.^19^^,^^20^

Among African Americans, increased susceptibility to POAG may also be influenced by the extent of African ancestry. On average, African Americans have roughly 20% European ancestry, although ancestry proportions can vary significantly among individuals.^21^ These varying proportions of ancestry have been shown to affect disease prevalence, severity, and resistance among populations. These differences are in part owing to variations in allele frequency, copy number variants, allelic architecture, and linkage disequilibrium among populations,^22^^–^^24^ which arose from the emergence of variants or natural selection in specific environments.^23^ For example, the International HapMap Project Phase 3 found that the risk loci for common complex diseases varied widely among 11 population groups.^25^ However, noninherited factors such as access to care, poverty, lifestyle, culture, or unmeasured environmental exposures (such as cumulative burden of racism) can also contribute to population differences in disease risk.^26^^–^^28^ An example of this relationship is asthma, which has a higher prevalence and severity in individuals of African descent. Both African genetic ancestry^29^ and external factors such as exposure to air pollution and allergens^30^^,^^31^ have been shown to contribute to higher asthma risk in individuals of African descent.

Although it is known that African Americans are disproportionately affected by POAG, the role genetics and particularly ancestry play in the disease's etiology and progression remains poorly understood after taking into account known risk alleles. Studies on POAG have primarily used self-reporting to define racial categorization, which is often an imprecise measure of genetic ancestry.^32^^,^^33^ The African Descent and Glaucoma Evaluation Study showed that higher African ancestry was associated with thinner corneas and larger discs,^34^ whereas a South African cohort found an association with both thinner corneas and increased IOP.^35^ Our previous study and other studies have also reported that specific mitochondrial haplogroups are associated with risk of POAG.^36^^,^^37^ However, aside from these studies, research using quantitative assessments of genetic ancestry remains limited for POAG.

The goal of this study was to investigate the association between genetic ancestry and POAG in a large, primary cohort of self-identified African Americans. These patients were recruited as part of the Primary Open-Angle African American Glaucoma Genetics (POAAGG) study, a 5-year project investigating the genetic architecture of POAG in self-identified African Americans. Using array-based genotyping data, clinically validated POAG diagnoses, and quantitative endophenotypic data, we conclude that average autosomal genetic ancestry may be a significant risk factor for POAG in African Americans,^38^ when not considering known nongenetic risk factors.

## Methods

### Subjects

The POAAGG study population consists of self-identified blacks (African American, African Caribbean, or African descent), aged 35 years or older. Subjects were identified from comprehensive and subspecialty ophthalmology clinics at the University of Pennsylvania and satellites, as well as several neighboring ophthalmology clinics in Philadelphia, Pennsylvania (Windell Murphy, MD, Temple University). Following examination by a fellowship-trained glaucoma specialist, each subject was classified as a POAG case, POAG suspect, or control based on previously reported criteria.^39^ In brief, cases were defined as having an open iridocorneal angle and characteristic optic nerve defects with corresponding visual field loss, whereas controls exhibited a lack of confounding ocular conditions. Information on eligibility criteria, study procedures, and phenotyping (including 97% concordance rates for defining cases and controls among glaucoma specialists across institutions) is provided elsewhere.^39^ The study protocol and consent statement were approved by the University of Pennsylvania Institutional Review Board.

### Specimen Collection and DNA Extraction

Genomic DNA for all enrolled subjects was extracted from peripheral blood or saliva, which both demonstrated excellent performance in array-based genotyping in a previous study.^40^ Blood was collected by venipuncture in 10-mL purple top tubes with EDTA anticoagulant. These samples were frozen at –20° before DNA isolation. For saliva collection, subjects were asked to refrain from smoking, drinking, or eating before donating specimens. Two milliliters of saliva per subject were collected in Oragene DISCOVER (OGR-500) self-collection kits (DNA Genotek Inc, Kanata, Ontario, Canada). The saliva specimens were mixed with stabilizing reagent within the collection tubes per manufacturer's instructions and stored at room temperature until DNA extraction.

DNA was isolated from thawed blood samples using Gentra PureGene kits (Qiagen, Valencia, CA) and included the optional RNase treatment step. DNA from saliva samples was extracted using the prepIT.L2P reagent (cat # PT-L2P-5, DNA Genotek Inc) and precipitated with ethanol according to manufacturer's instructions. The saliva DNA samples were RNAse treated by double digestion with RNase A and RNase T and reprecipitated using ethanol according to the manufacturer's instructions. The concentrations of DNA from blood and saliva samples were determined using the fluorescence-based Quant iT dsDNA Board-Range assay kit (cat # Q-33130, Life Technologies). Fluorescence was measured with a Tecan Infinite M 200 Pro multimode microplate reader (Tecan US, Inc, Raleigh NC).

### Genotyping and Quality Control (QC)

A 25-µL aliquot of all samples with high-quality DNAs and case/control status were plated for array-based high throughput genotyping. Genotyping was conducted on of 3830 DNA samples (1783 cases, 2047 controls) and a replication cohort of 2135 samples (755 cases, 1380 controls), using the Multi-Ethnic Genotyping Array (MEGA)V2 (EX) consortium chip on the Infinium iSelect platform by Illumina FastTrack Services (Illumina, San Diego, CA). The replication cohort consists of subjects who were enrolled after genotyping of the discovery samples was completed. The selection criteria for the samples, genotyping array, and QC were the same as for the discovery cohort.

MEGA array content was supplemented with 5000 single nucleotide polymorphisms (SNPs) from prior genome-wide association study (GWAS) on POAG, common polymorphisms from POAG-associated genes, and variants detected from whole-genome sequencing of POAAGG subjects. At least one sample per 96-well microtiter plate was genotyped in duplicate for purposes of QC. Genotype calls were generated using the Genome Studio genotyping module (GT). Cluster optimization and reproducibility analysis for paired samples were performed as per standard practices at Illumina FastTrack services.

Directly genotyped variants and samples were subjected to rigorous QC.^41^ First, samples were removed that had discordant genders, outlying heterozygosity (±3 sigma), or at least 3% missing genotype calls. Next, identity-by-descent matrix calculations with PLINK were used to remove one of any pair of samples with an identical by descent of 0.1875 or greater (Supplementary Fig. S1, Supplementary Table S1). A subset of samples were excluded from ancestry analysis after QC owing to missing data. After this individual-level QC, variants were removed from further consideration by the following criteria: more than 3% of samples with missing calls, statistically significant evidence of differential call rate between cases and controls, deviation from Hardy–Weinberg equilibrium at a significance level at a *P* value of less than 0.00001, and a minor allele frequency of less than 0.01 (rare variants) (Supplementary Fig. S2).

### Ancestry Analysis

After individual-level and variant-level QC, 1,108,459 SNPs were analyzed for continental ancestry using the 1000 Genomes Project version 5a dataset. This dataset contains 2506 human samples from 26 global populations grouped into five labeled superpopulations (African, Admixed American, East Asian, European, and South Asian). We removed African American samples from the African superpopulation to achieve a higher resolution of African–European admixture. After removing A->T/T->A/C->G/G->C genetic variants for strand alignment, autosomal genotypes were merged between the POAAGG cohort and the 1000 Genomes Project dataset before LD pruning at an R2 ceiling of 0.2 before principal components from a genetic relationship matrix were extracted using Plink version 1.90.

We next investigated two-way admixture among cases and controls using fastSTRUCTURE, which is a software designed to infer population structure from genotyped data.^42^ The fastSTRUCTURE program was used to estimate the proportion of two ancestral populations in POAAGG cases and controls, yielding q0 and q1 values for each study sample. Autosomal genetic ancestry was estimated using two ancestral populations and parameters “–full –seed 777”. This analysis generated two ancestral components (q0 and q1), which sum to 1.0 and represent the fraction of ancestry derived from two ancestry populations. We oriented these two ancestral proportions by comparing q0 with the PC1 variable derived from continental ancestry analysis by principal components.

To quantify the relationship between African ancestry and case/control status, we divided the q0 variable into quartiles. The lowest quartile of q0 was associated with the highest degree of African ancestry and the lowest degree of European ancestry under the two-way admixture model from fastSTRUCTURE.

### Quantitative Endophenotypes

We used logistic regression models that adjusted for age and gender to calculate the odds ratio and its 95% confidence interval for the association between the estimated African ancestry from fastSTRUCTURE (q0) and POAG. Among POAG cases, we used linear regression models that adjusted for age and gender to determine the associations between African ancestry and four quantitative phenotypes: IOP, central corneal thickness (CCT), cup-to-disc ratio, and retinal nerve fiber layer thickness.

### Local Admixture Analysis

To perform admixture mapping in the POAAGG samples, we used ANCESTRYMAP, which estimates the ancestral origin of a locus and then tests whether there is a skew in ancestry associated with case status at each marker.^43^ We filtered the genotypes to consider only 2539 loci that are known to be associated with African ancestry.^44^ This analysis was run on 3978 cases with nonmissing phenotype information and who passed sample QC, and the analysis was adjusted for age and sex as covariates. Variants were considered significant if the *P* value passed a Bonferroni-corrected threshold of 1.96^–^^5^.

### Polygenic Gene Risk Score

Prior POAG-associated variants were extracted from the NHGRI-EBI GWAS catalog,^45^ filtered for “primary open angle glaucoma” in the trait column. Studies including at least some African and/or European ancestry individuals were included and duplicate genetic variants were removed, leaving 23 previously published SNPs that were genotyped in the POAAGG study (Table 4). SNP genotypes were extracted with PLINK and a weighted polygenic risk score was computed as the sum of the risk allele count times the published odds ratio for that risk allele.^45^

### Multivariable Risk Model

To develop a multivariable risk model, a logistic regression model was fit on the whole cohort using age, gender, polygenic gene risk score, and ancestry as predictors. The association of each predictor with POAG risk was assessed by odds ratios (95% confidence intervals) and *P* values.

## Results

### African Ancestry and POAG Association

**Figure 1.**
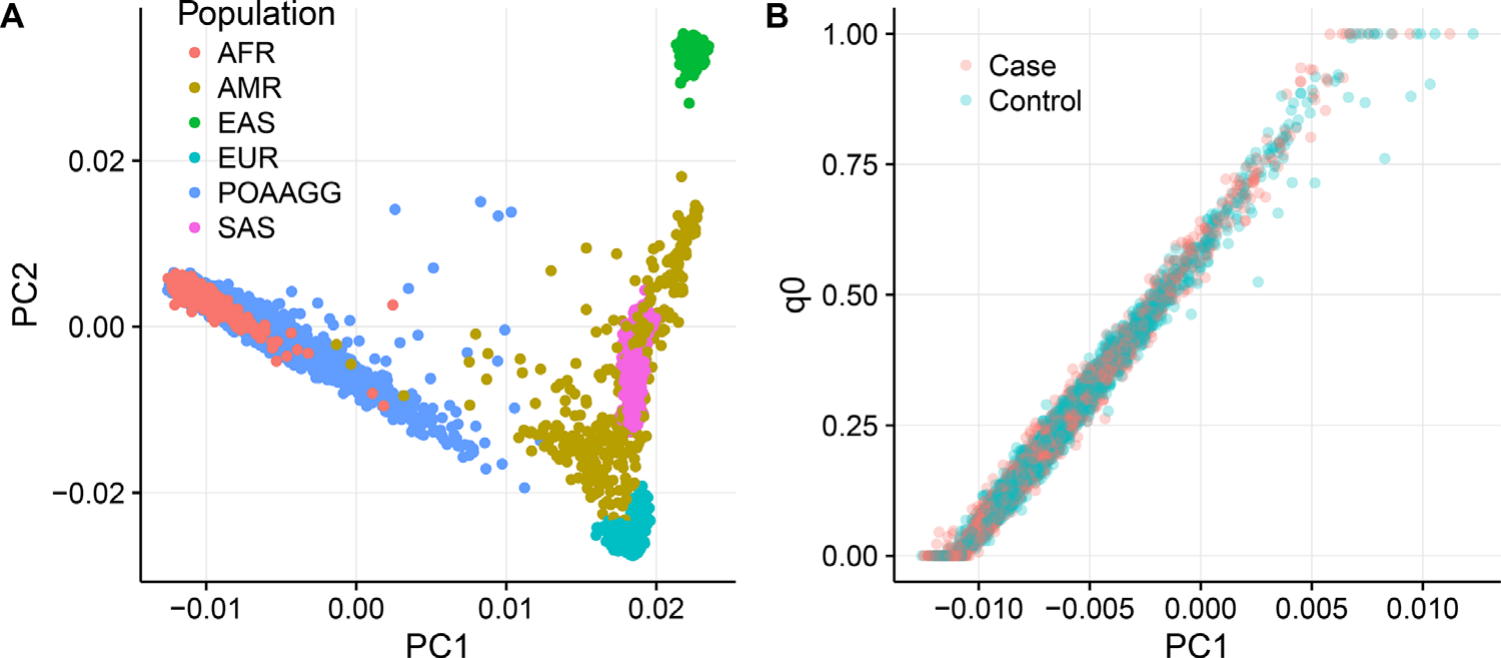
Inferred African ancestry in POAAGG study samples, stratified by case status. (**A**) PC1 vs. PC2 scatterplot for cases and controls after merging with 1000 Genomes Project. LD-pruned genotypes from the 1000 Genomes Project, excluding the African American (ASW) subpopulation from the 1000 Genomes Project, were merged with POAAGG genotypes and principal components were computed from a genetic relationship matrix. Continental ancestry for the POAAGG cohort is visualized in PC1 versus PC2 space. PC1 is found to separate AFR (African) from EUR (European) samples within the 1000 Genomes Project; (**B**) Genome-wide admixture analysis with fastSTRUCTURE compared to PC1. Proportion of two-way admixture among autosomal genotypes was estimated using fastSTRUCTURE software with two ancestral populations (k = 2) using only POAAGG cases and controls. Output from fastSTRUCTURE represents the proportion of genotypes derived from each of two ancestral populations: q0 and q1. To orient q0 with respect to principal components, PC1 is plotted against q0. This component, q0, takes the highest values in genomes with the highest values of PC1 (as in Fig. 1A) which is consistent with increased African ancestry.

The majority of POAAGG samples lie on an axis between the African and European 1000 Genomes reference samples, with a high degree of variation in ancestry proportions among individuals (Fig. 1A). The first principal component (PC1) separates African and non-African reference populations in this analysis (Fig. 1A).

For the subsequent ancestry analysis, a small subset of samples were excluded after QC owing to missing data, resulting in a discovery cohort of 3806 patients and replication cohort of 2011 patients. For the discovery cohort (*n* = 3806), glaucoma cases had significantly lower mean values of PC1 compared with controls, consistent with a higher degree of African ancestry (*P* = 5^–^^6^; Table 1). This ancestry effect was also highly significant in the replication cohort (*n* = 2011; *P* = 5^–^^6^).

**Table 1.** The Association of Independent Metrics of African Ancestry with POAG Risk in Discovery Cohort and Replication Cohort

Ancestry Metric	Controls: Mean (SD)	Cases: Mean (SD)	Adjusted OR for One SD Increase in Ancestry Metric (95% CI)	Adjusted *P* Value
Discovery cohort				
PC1 (n = 3806)	−0.0069 (0.0036)	−0.0074 (0.0036)	0.85 (0.79–0.91)	0.000005
q0 (n = 3413)	0.27 (0.18)	0.25 (0.19)	0.88 (0.82–0.95)	0.000614
Replication cohort				
PC1 (n = 2011)	−0.0103 (0.0038)	−0.0111 (0.0036)	0.79 (0.71–0.87)	0.000005
q0 (n = 2011)	0.26 (0.19)	0.22 (0.18)	0.79 (0.71–0.87)	0.000003

CI, confidence interval; OR, odds ratio; SD, standard deviation.

Two ancestry metrics, PC1 from principal components analysis and q0 from fastSTRUCTURE, were computed for POAG cases and controls. Mean values in cases and controls and standard deviation (SD) were included. Adjusted odds ratio were estimated from separate logistic regression models (one for PC1, another for q0) and adjusted for gender and age at enrollment. PC1 and q0 scores were standardized for computing odds ratios, allowing comparison between PC1 and q0, which have different mean and range.

When orienting two ancestral proportions (q0 derived from fastSTRUCTURE, PC1 derived from continental ancestry analysis by principal components), we found that q0 and PC1 were highly correlated (r = 0.991; *P* < 0.001; Fig. 1B). The mean q0 was lower among cases than controls in both the discovery cohort (*P* = 6.14^–^^4^) and replication cohort (*P* = 3^–^^6^) suggesting a higher degree of African ancestry in cases (Table 1). Thus, we use q0 as the genetic ancestry value for each participant in subsequent analyses.

We divided the q0 estimate into quartiles, with the lowest quartile of q0 indicating high African and low European ancestry (Table 2). The adjusted odds of being a case were slightly lower in the third and fourth quartiles, which have the lowest degree of African ancestry. The same suggestive effect was seen in the replication cohort.

**Table 2.** Case/Control Status, Mean Age, and Sex Tabulated by Quartiles of Autosomal Ancestry in the Discovery Cohort

Ancestry Quartile (q0)	*N*	Males (%)	Mean Age in Years (SD)	Cases (%)	Adjusted OR for Case Status (95% CI)
Discovery cohort					
(0.000,0.125)	853	320 (38)	65.8 (12.4)	451 (53)	Reference
(0.125,0.223)	853	286 (34)	64.9 (11.9)	394 (46)	0.79 (0.65–0.97)
(0.224,0.350)	854	288 (34)	63.8 (12.2)	357 (42)	0.69 (0.56–0.85)
(0.350,1.000]	853	299 (35)	65.6 (12.6)	383 (45)	0.70 (0.57–0.86)
Replication cohort					
(0.000,0.106)	502	190 (38)	65.6 (11.5)	214 (43)	Reference
(0.106,0.219)	503	174 (35)	64.0 (11.4)	185 (37)	0.86 (0.66–1.13)
(0.219,0.345)	503	170 (34)	63.1 (11.2)	158 (31)	0.70 (0.54–0.92)
(0.345,1.000]	503	156 (31)	65.4 (12.7)	146 (29)	0.54 (0.41–0.71)

Estimated African ancestry by fastSTRUCTURE (q0) was grouped into quartiles. Percent of cases and males were tabulated for each quartile, and mean and standard deviation (SD) of age was computed. Odds ratios for POAG by quartiles of q0 were calculated from logistic regression model adjusting for age and sex.

### Quantitative Endophenotypic Analysis

We observed a significant relationship between African ancestry and thinner CCT in both discovery cohort (*P* < 0.001) and replication cohort (*P* = 0.002), as shown in Table 3. The association between African ancestry and IOP was significant, but only in the replication cohort (*P* = 0.02). The other quantitative traits (cup-to-disc ratio, retinal nerve fiber layer mean thickness) were not significantly associated with African ancestry.

**Table 3.** Linear Regression of Estimated African Ancestry (q0 Estimate From fastSTRUCTURE) Effect on Four Quantitative Traits Among POAG Cases

	Discovery Cohort			Replication Cohort		
Quantitative Trait	No. of Cases with Measure for Analysis	Regression Coefficient for African Ancestry (95% CI)	*P* Value	No. of Cases with Measure for Analysis	Regression Coefficient for African Ancestry (95% CI)	*P* Value
IOP (mm HG)	1336	–0.08 (–0.31 to 0.16)	0.52	559	–0.45 (–0.85 to –0.06)	0.02
CCT (microns)	1503	3.93 (2.03 to 5.82)	<0.001	484	5.51 (2.10 to 8.91)	0.002
CDR	1527	–0.004 (–0.012 to 0.004)	0.36	520	–0.005 (–0.019 to 0.009)	0.50
RNFL mean thickness (microns)	1134	0.46 (–0.28 to 1.19)	0.22	471	0.51 (–0.65 to 1.67)	0.39

CDR, cup-to-disc ratio; RNFL, retinal nerve fiber layer. For each trait, a multivariable linear model was constructed using the standardized q0 estimate (African ancestry) as a predictor, adjusting for age and sex and the *P* Values and estimated beta coefficient of the q0 term (along with 95% CI) are reported.

### Local Admixture Analysis

**Figure 2.**
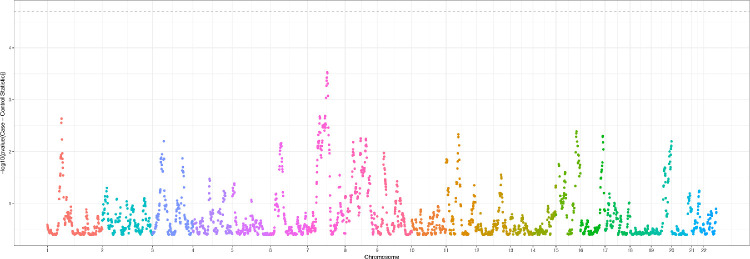
Manhattan plot of admixture mapping results using ANCESTRYMAP. The -log10(*P* value) for each of 2,539 African ancestry informative markers testing the association of African ancestry with case status is plotted. The dotted gray line indicates genome-wide significance, here a Bonferroni-corrected threshold of 1.96e-05. No individual variant reached significance.

We performed local admixture mapping with the ANCESTRYMAP software.^43^ The PC1 values had a Pearson correlation coefficient of 0.988 with the average proportion African ancestry estimated with ANCESTRYMAP, ensuring that the ANCESTRYMAP proportions are commensurate with genetic ancestry estimated when using reference samples. We tested for an association between case status and African ancestry at each locus, adjusting for age and sex. The Manhattan plot shows that no single variant was significantly associated with case status (Fig. 2). We did not perform replication analysis for the admixture mapping owing to a lack of significant findings in the discovery cohort.

### Gene Risk Score and Multivariable Risk Model

We constructed a polygenic risk score from 23 glaucoma-associated SNPs from the NHGRI-EBI GWAS catalog (Table 4). This risk score was associated significantly with POAG in both the discovery cohort (*P* < 0.001) and replication cohort (*P* < 0.001).

**Table 4.** Prior POAG-Associated SNPs Used to Construct a Weighted Polygenic Gene Risk Score

rsID	Risk Allele	Directly Genotyped	Chro-mosome	Position	Reported Gene(s)	OR	African Risk Allele Frequency	European Risk Allele Frequency
rs2472493	G	Yes	9	104933567	*ABCA1*	1.31	0.311	0.418
rs4619890	G	Yes	4	7851433	*AFAP1*	1.20	0.859	0.466
rs2276035	A	Yes	11	120475651	*ARHGEF12*	1.18	0.251	0.164
rs7137828	T	No	12	111494996	*ATXN2*	1.17	0.981	0.536
exm-rs4236601	A	Yes	7	116522675	*CAV1, CAV2*	1.27	0.399	0.259
rs4977756	A	Yes	9	22068653	*CDKN2B-AS1*	1.48	0.68	0.6
rs9475699	A	Yes	6	56437256	*COL21A1-DST*	1.04	0.228	0
rs56962872	G	No	3	186482434	*CRYGS, LINC02052, TBCCD1*	1.14	0.933	0.69
rs2073006	T	No	6	637465	*EXOC2*	1.16	0.045	0.131
rs2745572	A	No	6	1548134	*FOXC1*	1.22	0.85	0.636
seq-rs9913911	A	Yes	17	10127866	*GAS7*	1.17	0.836	0.627
seq-rs11969985	G	Yes	6	1922673	*GMDS*	1.31	0.741	0.851
rs56335522	G	No	2	212893510	*IKZF2*	1.18	0.904	0.878
rs2710323	T	Yes	3	52781889	*ITIH1*	1.14	0.278	0.495
rs9530458	T	No	13	75675139	*LMO7*	1.148	0.359	0.527
rs6478746	G	No	9	126605119	*LOC105376277, LMX1B*	1.14	0.266	0.297
rs4918865	C	No	10	93183256	*MYOF, XRCC6P1, CYP26A1, CYP26C1, EXOC6*	1.119	0.635	0.594
rs9494457	T	No	6	136153656	*PDE7B*	1.08	0.629	0.633
rs10483727	T	Yes	14	60606157	*SIX6*	1.32	0.966	0.404
rs4656461	G	Yes	1	165717968	*TMCO1*	1.38	0.256	0.141
rs35934224	C	No	22	19885122	*TXNRD2*	1.28	0.691	0.846
rs2041895	C	No	12	106956310	TMEM263*	1.48	0.177	0.537
rs284491	T	No	8	104946405	None	1.52	0.719	0.659

However, when adjusted for this polygenic effect, African ancestry remained as a significant risk factor (Table 5). This finding was validated in our replication cohort. Advanced age and male sex were also strong risk factors for disease (*P* < 0.001 for both).

**Table 5.** Multivariable Risk Model^*^ of POAG in African Americans

	Discovery Cohort		Replication Cohort	
	OR (95% CI)	*P* Value	OR (95% CI)	*P* Value
Age (per 10 years increase)	2.03 (1.90–2.18)	<0.001	1.86 (1.70–2.04)	<0.001
Ancestry (q0) (per 1 SD increase)	0.90 (0.83–0.97)	0.004	0.82 (0.74–0.91)	<0.001
Gene risk score (per one risk point increase)	1.08 (1.06–1.11)	<0.001	1.08 (1.05–1.12)	<0.001
Male sex	1.57 (1.35–1.83)	<0.001	2.12 (1.73–2.61)	<0.001

* Multivariable model included age, ancestry (q0), gene risk score and sex as predictors.

## Discussion

We assessed the association of autosomal genetic ancestry with POAG risk in a large self-identified African American cohort, performing global and local ancestry analyses estimated from array-based genotyping data. We found that a higher proportion of African ancestry in the nuclear genome increased disease risk for subjects, whereas local genetic ancestry was not a significant risk factor, when only considering genetic risk factors for POAG.

In this study, the proportion of African ancestry varied greatly in our cohort, consistent with prior studies.^21^^,^^46^ African populations have greater genetic diversity than any other population,^24^^,^^47^^–^^49^ with more haplotypes, more complex patterns of population substructure, and weaker linkage disequilibrium than other groups.^24^^,^^49^ African Americans were recently admixed within the past 20 generations.^50^ Today, this population has an average of 20% European ancestry, although ancestry proportions vary substantially among individuals, as seen in our cohort.^21^ One previous study showed that African Americans from Philadelphia have approximately 12% to 13% European ancestral proportions, but no other studies have examined this city to our knowledge.^46^

We found that, without considering nongenetic risk factors, genome-wide African ancestry is a risk factor for POAG in our African American cohort. Although many previous studies have shown that individuals of African descent are disproportionately affected by POAG, this study used a quantitative assessment of ancestry rather than self-reporting as an independent variable. An empirical assessment of genetic ancestry is a more precise measure of biologic diversity, allowing analyses of continuous rather than a categorical variables.^32^ Our results show that there are varying degrees of disease risk within the African American population, based on an individual's specific ancestral proportions. Two independent approaches support this finding. First, principal components analysis was performed, a technique that uses an ancestry-labeled population panel (The Thousand Genomes Project). Second, autosome-wide ancestry mapping with fastSTRUCTURE was used to directly infer ancestral components from a two-way admixture model using the cohort itself, without regard to an external panel. Local admixture mapping showed that this is a global genomic effect, with no specific loci achieving a significant association.

Consistent with prior studies, greater African ancestry was associated with thinner CCT. It is well-established that healthy African Americans have a thinner CCT than healthy European Americans.^51^^,^^52^ However, previous studies are divided on whether or not thin CCT is a predictive factor for POAG development.^35^^,^^53^^–^^55^ Some results support the conclusion that thinner CCT can lead to the underestimation of IOP, as the Goldmann applanation technique (commonly used to measure IOP) assumes that CCT does not vary significantly between individuals. It is possible that this underestimation of IOP could lead to undertreatment of disease and increased optic nerve damage in African Americans.^52^

We also tested the significance of a polygenic gene risk score in our cohort, with results showing a significant polygenic effect with regard to disease risk, independent of ancestry. This finding validates the additive effect of known POAG-associated loci in our African American population. Most recently, a GWAS identified a novel locus in *APBB2* as significantly associated with POAG in individuals of African ancestry.^56^ Our studies to assess the polygenic risk score were performed before the publication of this article. To evaluate the inclusion of *APBB2* in our polygenic risk score, we attempted to replicate the *APBB2* (rs59892895) variant in our case-control POAAGG study and found that the association was not significant (*P* = 0.335) (data not shown, Gudiseva et al., 2020, doi:https://doi.org/10.1101/2020.02.27.968156). These results suggest that inclusion of the novel *APBB2* variant would not affect the polygenic risk score for this highly admixed African American population. Further studies with the Genetics of Glaucoma in People of African Descent (GGLAD) Consortium are currently underway to better define genetic similarities and differences between these populations. When examining the multivariable risk model, African ancestry remains as a significant risk factor when adjusted for this polygenic effect, a finding that was validated in our replication cohort. POAG risk in African Americans is likely influenced by the additive effects of polygenic risk at known disease-associated loci and an omnigenic ancestry effect.

The strengths of this study include the use of a genotyping array specifically designed to maximize coverage of genetic variants relevant to African Americans. This custom design may help to avoid the limitations of previous studies that used arrays designed for European populations on African populations, leaving up to 40% of variants not assayed in African populations.^57^^,^^58^ We also recruited our entire cohort from Philadelphia, allowing us to control for differences in population structure, environment, and socioeconomic factors. We performed the analyses in a discovery cohort and replication cohort separately to validate the study findings. In addition, we carefully ascertained phenotypes from every subject, which is crucial to prevent residual confounding effects of unmeasured phenotypes within association studies.^59^

This study does not address how genetic risk factors interact with social and demographic variables to affect POAG risk in African Americans. Prior studies have shown that social and demographic factors interact with biological factors to affect health outcomes.^33^ These factors can include (but are not limited to) educational attainment, income, socioeconomic status, social and cultural identity, and other dimensions of race (such as cumulative burden of discrimination.^33^ We do not have individual-level data on these factors for our subjects and thus were not able to explore these complex relationships in this study. These confounders cannot be ruled out as contributors to elevated POAG risk in individuals of African descent. The absence of an association with African-specific alleles in the admixture analysis further suggests that environmental and social factors, in addition to African ancestry, may contribute to POAG risk. In the future, our study has the unique ability to recontact patients to collect demographic information and further explore these questions. Our entire cohort was recruited in a single city and focused heavily on community engagement,^39^^,^^60^^,^^61^ allowing us to bring families back and screen at-risk populations as we continue to investigate this disease.

In summary, we found that higher levels of autosomal-wide African ancestry serve as a risk factor for POAG in self-identified African Americans. Although global ancestry was highly predictive of POAG risk, no specific African ancestry informative markers rose to genome-wide significance. This study illustrates the great genetic diversity among African Americans and the importance of using quantitative assessments of ancestry when the aim is to identify genetic associations. In future studies, it will be important to capture and explore how social and environmental variables interact with genetic risk factors to affect POAG risk. We hope that studying this admixed population will be helpful in mapping POAG genes with prevalence differences between ethnic groups. Furthermore, the finding that the highest degree of African ancestry conferred the greatest POAG risk suggests ancestry analysis could contribute to precision screening of this population.^62^

## Supplementary Material

Supplement 1
