# Supplementary material for: The Role of Genetic Ancestry as a Risk Factor for Primary Open-angle Glaucoma in African Americans
Source: Invest Ophthalmol Vis Sci. 2021 Feb 19;62(2):28. doi: 10.1167/iovs.62.2.28 (PMC7900887; doi:10.1167/iovs.62.2.28)
Supplement: Supplement 1 [file iovs-62-2-28_s001.pdf]

Supplementary Information

Supplementary Figure S1: Quality Control Flowchart for Ancestry Analysis of Discovery Cohort.

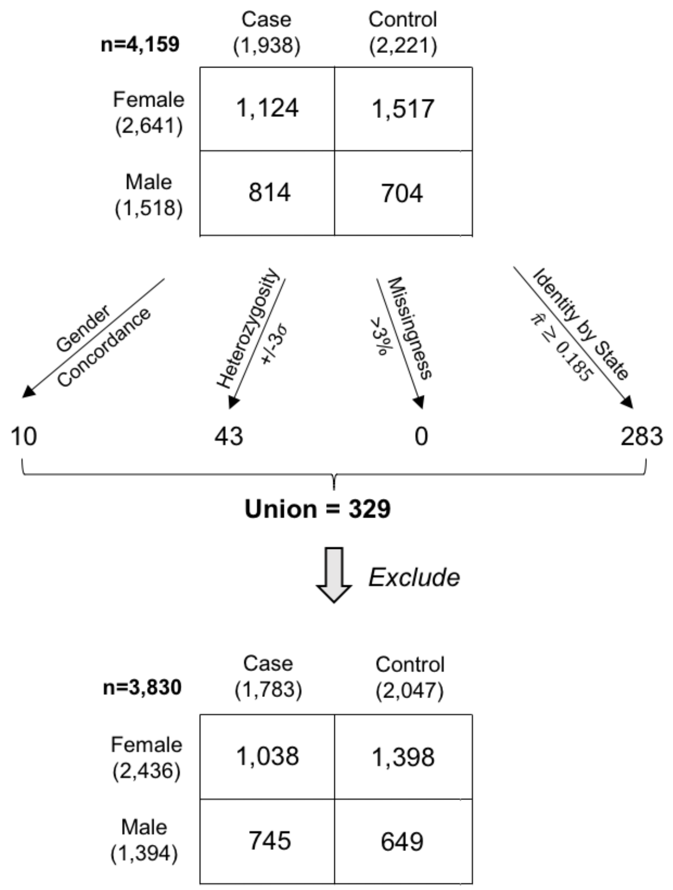

Ancestry analysis QC pipeline excluded one sample in pairs of related individuals and those with outlying heterozygosity. Variant-level QC was applied to eliminate variants with differential missingness between cases and controls, genotype missing rate greater than 3% across samples that survived individual-level QC, departures from Hardy-Weinberg Equilibrium, and variants that were rarer than 1% minor allele frequency. The resulting 1,108,459 genetic variants were used for ancestry analysis by PCA and fastStructure, with LD pruning threshold r^2 of 0.2. QC and pruning were performed with PLINK version 1.9 and custom codes.

Supplementary Figure S2: Quality Control Flowchart at Variant Level for Ancestry Analysis.

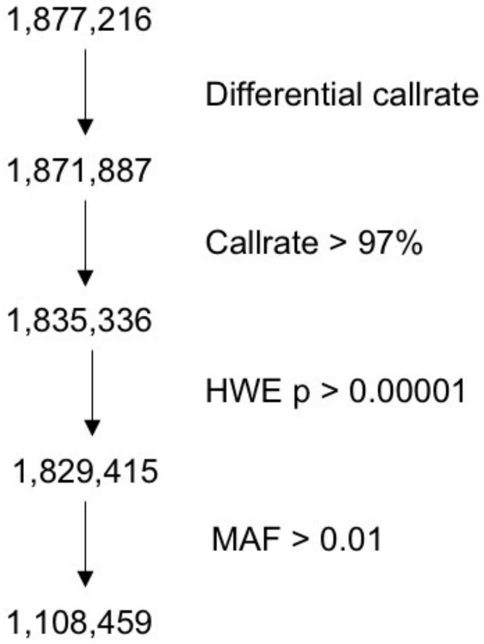

Supplementary Table S1: Quality Control Flowchart for Ancestry Analysis of Replication Cohort.

Step number	Step name	SNP count (BIM file)	Sample count (FAM file)
0	Initial dataset	2040811	2245
1	Sex check	2040811	2238
2	Marker callrate	1864749	2238
3	Sample callrate	1864749	2238
4	MAF	1139520	2238
5	IBD	1139520	2160
6	Heterozygosity	1139520	2135
7	Marker callrate	1139376	2135
8	Sample callrate	1139376	2135
9	MAF	1137606	2135
10	HWE	1127544	2135
11	Differential missingness	1127528	2135
